# Mr. Parker

**Published:** 1825-01-01

**Authors:** 


### MR. PARKER.

This Gentleman, who was likely to prove an ornament to the Profession, by his talents and acquirements, suddenly expired on Monday, the 6th of December, at his house in Devonshire Street. He was in perfect health, and in consultation with the Editor of this Journal, at 12 o'clock of the day he died. At 5 o'clock, he took 25 drops of laudanum and retired to bed, complaining of head-ache, as he had been called out the night before. At nine o'clock, he was found quite cold and lifeless; so that he must have expired shortly after he retired to his bed. On examination, it was found that he died of sanguineous apoplexy.

